# Analysis of leaf forage value and screening of different populations of *Pteroceltis tatarinowii*, a rare and endemic species in China

**DOI:** 10.3389/fpls.2023.1164451

**Published:** 2023-06-30

**Authors:** Yun Gao, Tian-Tian Cheng, Chun-Xiang Zhang, Yu Yan, Lin Zhang, Qing-Zhong Liu, Yan Liu, Qian Qiao

**Affiliations:** ^1^ College of Plant Protection, Shandong Agricultural University, Tai′an, China; ^2^ Shandong Institute of Pomology, Shandong Academy of Agricultural Sciences, Tai′an, China; ^3^ Taishan Forestry Science Institute, Tai′an, China; ^4^ College of Landscape Architecture, Beijing Forestry University, Beijing, China

**Keywords:** *Pteroceltis tatarinowii*, woody forage, crude protein (CP), forage value, population diversity

## Abstract

To fully exploit the economic value of the Chinese endemic species *Pteroceltis tatarinowii* and provide new resources for forage production, the forage nutritional value of *P. tatarinowii* leaves from different populations was analyzed and evaluated. The results were as follows: 1) There were significant differences in the forage nutrient indices of leaves from different populations. The crude protein content was 10.77%–18.65%, with an average of 14.58%, and the SDJN population had the highest crude protein content. The average crude fat, crude fiber content was 7.62%; the average neutral detergent fiber content was 25.33%; and the average acid detergent fiber contents were 6.79%, 7.62%, 25.33%, and 17.52%, respectively. The average phosphorus and calcium content in the leaves was 0.785 g·kg^−1^ and 58.01 g·kg^−1^, respectively. The tannin content was much lower than the antifeedant standard, at an average of 4.97 g·kg^−1^. The average total amounts of hydrolyzed and free amino acids in the leaves were 108.20 mg·g^−1^ and 47.87 mg·g^−1^, respectively. Thus, *P. tatarinowii* leaves have high crude protein, crude fat, and calcium contents, and low fiber, tannin contents, and are protein-rich. These results provide evidence that this species can be developed into an excellent woody forage tree. 2) There were significant differences in the forage quality evaluation indices among the populations. The forage indices of NDP, ADP, DMI, DDM, and RFV of 21 populations all met the super standard of the American Grass and Grassland Association (AFGC) for hay, two crude protein indices met the grade 1 standard, and 12 crude protein indices met the grade 2 standard. Four high-protein and high-RFV forage populations (SDJN, SDZZ, SXLQ, and AHXX) were selected. 3) The results of the correlation analysis showed that there was no significant correlation between the forage characteristics of *P. tatarinowii* leaves and latitude and longitude, indicating no significant geographical variation. However, the forage characteristics were strongly correlated with elevation, average annual temperature, and annual precipitation. Thus, high elevation, low temperatures, and rainy weather can improve the forage value of the leaves. *P. tatarinowii* can be planted to provide leaf forage in cold and wet areas at a specific elevation. Moreover, the forage value of *P. tatarinowii* leaves can be further improved by increasing nitrogen fertilizer and reducing K and Ca fertilizers during cultivation. 4) Cluster analysis revealed obvious regionalism. Taking the Yangtze River Basin as the limit, cluster analysis divided the species into four population groups: the Yangtze River Basin and northern, southwestern, and eastern coastal populations.

## Introduction

Woody forage refers to young branches, leaves, flowers, fruits, seeds, and byproducts of woody plants with forage value that can be used directly for grazing, collecting, cutting, and processing. Compared with herbaceous forage, woody forage has the advantages of being highly nutritious, having a high crude protein content and high yields, occurring broadly, and being able to be used for many years (Moleele et al., 1998; Sliva et al., 2015). Simultaneously, woody forage does not compete with agricultural land. In today’s world, where pasture areas are shrinking and pasture degradation is serious, the development of woody forage is inexpensive with substantial benefits, and it does not occupy arable land. Additionally, it is important to address the issues of humans and animals competing for grain.

In recent years, countries worldwide have continuously promulgated regulations that prohibit growth-promoting drugs and additives in feed (Zhang et al., 2018). Woody forage has become a popular topic in feed resource development, and research on its development and utilization of woody forage is increasing. In South Asia, Southeast Asia, and many African countries, woody forage is an important feed resource that plays an important role in animal husbandry (Larbi et al., 1996; Argel et al., 2000; Franzel and Wambugu, 2007). Woody forage has been widely used as a nitrogen source for ruminants in hot and humid climates such as Latin America, Southeast Asia, and South Africa (Ibrahim et al., 2001; Jank et al., 2005; Shelton et al., 2005; Sánchez and Ledin, 2006). In India and Nepal, woody forage has become the most important component of agroforestry operations, accounting for 40% of the feed supply provided by woody forage (Pandey, 1982). In Thailand, cassava and pigeon pea are fed together with straw as forage for livestock (Vongsamphanh et al., 2016). In Costa Rica, *Cratylia argentea* is mixed directly with sugarcane or *Pennisetum sinese* or fed as silage to cows to reduce the cost of feed during the dry season (Argel et al., 2000). Robinia and poplar leaves have been used to produce livestock roughage in South Korea (Burner et al., 2008). In eastern Africa, *Calliandra haematocephala*, *Morus alba*, and plants of the genera *Leucaena* and *Sesbania* are widely used as fodders (Franzel and Wambugu, 2007). The woody forage plants developed in northern Africa are mainly *Cacti*, *Saltbushes*, and *Wattles* (Houerou, 2000). *Chamaecytisus palmensis*, *Leucaena leucocephala*, and *Atriplex patens* are used as woody forage plants in Australia (Lefroy, 2002; Vandermeulen et al., 2018). In the Americas, the main goal of woody forage research is to increase livestock productivity and improve forage quality. *Bauhinia purpurea*, *C. argentea*, *Flemingia macrophylla*, *Gliricidia sepium*, *Desmodium*, *Albizia*, and *Leucaena* have also been used as woody forage plants (Ibrahim et al., 2001; Jank et al., 2005; Shelton et al., 2005; Sánchez and Ledin, 2006). Woody species play an important role in feeding livestock in many European regions (Vandermeulen et al., 2018). Hejcman et al. (2016) measured and compared the forage characteristics of *Betula nana*, *Betula pubescens*, *Salix lanata*, *Salix phylicifolia*, and *Sorbus aucuparia* in Iceland. Papachristou and Papanastasis (1994) measured the crude protein content, neutral detergent fiber (NDF), lignin, and *in vitro* organic matter digestibility (IVOMD) of common woody forage species (e.g., *Robinia pseudoacacia*, *Amorpha fruticosa*, and *Colutea arborescens*) in different seasons in Greece. Ainalis and Tsiouvaras (1998) explored forage production of four woody fodder species and herbaceous vegetation in relation to plant spacing and animal (sheep) grazing. Hejcmanová et al. (2014) investigated the leaf forage quality of major broad-leaved wood species in Central Europe, and the results showed that species with low-forage-quality leaves were *Carpinus betulus*, *Fagus sylvatica*, and *Quercus robur*; species with intermediate-quality leaves were *Corylus avellana and Populus tremula*; and species with high-quality leaves were *Ulmus glabra*, *Fraxinus excelsior*, *Tilia cordata*, and *Acer platanoides*.

**Figure 1:**
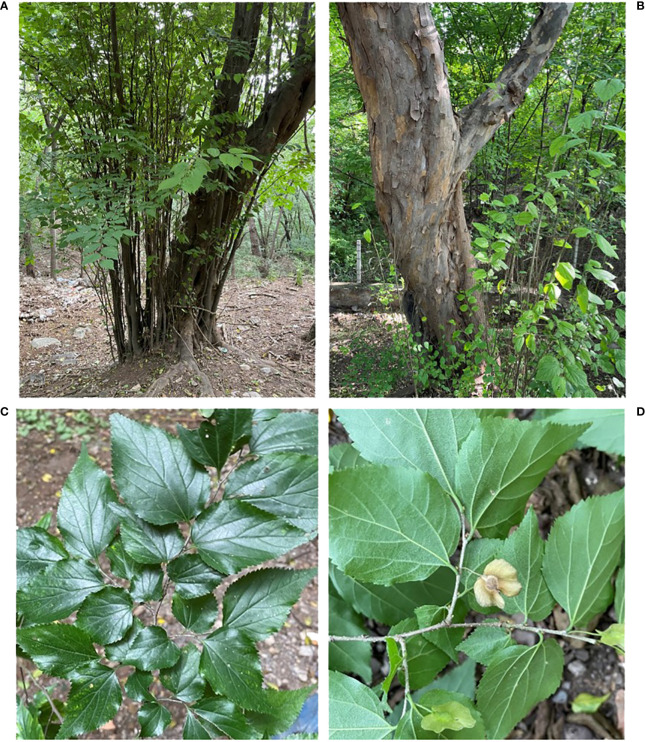
Photographs of *P. tatarinowii.* **(A)** Mature tree. **(B)** Bark. **(C)** Leaf. **(D)** Mature fruits.

Some woody forage plants materials have also been developed in China, such as the branches, leaves, and fruits of the Salicaceae family; the fruits of the Fagaceae family; the leaves of the Ulmaceae family; the leaves of the Moraceae family; the leaves and fruits of the Rosaceae family; the leaves and fruits of the Fabaceae family; the seeds of the *Acer* genus; the branches, leaves, and fruits of the Anacardiaceae family; the fruits and leaves of the Vitaceae family; the leaves of the Elaeagnaceae family; the pine needles of the Pinaceae family; the seeds of the *Sapindus* genus; leaves and inflorescences of the Juglandaceae family; the whole plant of the *Smilax* genus, and the leaves of the Bambusoideae family. As one of the most abundant plant species in the world, China has more than 8,000 woody plant species, with numerous resources that can be used for woody forage. Our research group has preliminarily confirmed that *Pteroceltis tatarinowii*, belonging to the Ulmaceae family, has the potential to become a woody forage, and the crude protein content of its leaves is 17.60%. It has high crude protein, crude fat, and calcium contents and low crude fiber, neutral detergent fiber, acid detergent fiber, and tannin contents. The deciduous tree species of the Ulmaceae family, *P. tatarinowii* Maxim. is the only plant in the genus *Pteroceltis* and has been listed as a national third-level key protection object in the Chinese Plant Red Book. In addition, this species is an important indicator plant of fibrous tree species and calcareous soil endemic to China, and is an important economic tree species, as well as an important native tree species and garden greening tree species (**Figure 1**). *P. tatarinowii* has strong adaptability and affinity for calcium, growing on limestone mountain, and can also grow in areas dominated by granite, and sandstone area. It is resistant to drought and barren environments, has a developed root system, grows by tillering, has a long life, and other excellent characteristics. It is widely distributed in North, East, Central, and South China and is distributed sporadically in 19 provinces and cities in China (Chai et al., 2010; Li et al., 2012; Liu et al., 2019); however, differences in forage characteristics in different regions have not been studied. Therefore, further develop and utilize the forage values of *P. tatarinowii* leaves, the forage characteristics of *P. tatarinowii* leaves from different populations were evaluated, to determine the differences among populations and the geographical variation trends; identify the superior populations; lay the foundation for breeding, developing, and using the superior *P. tatarinowii* for forage; and further improve the value added by *P. tatarinowii* cultivation. This study also provides a foundation for the further collection and protection of wild *P. tatarinowii* germplasm resources.

## Materials and methods

### Test materials

#### Leaf collection

**Figure 2:**
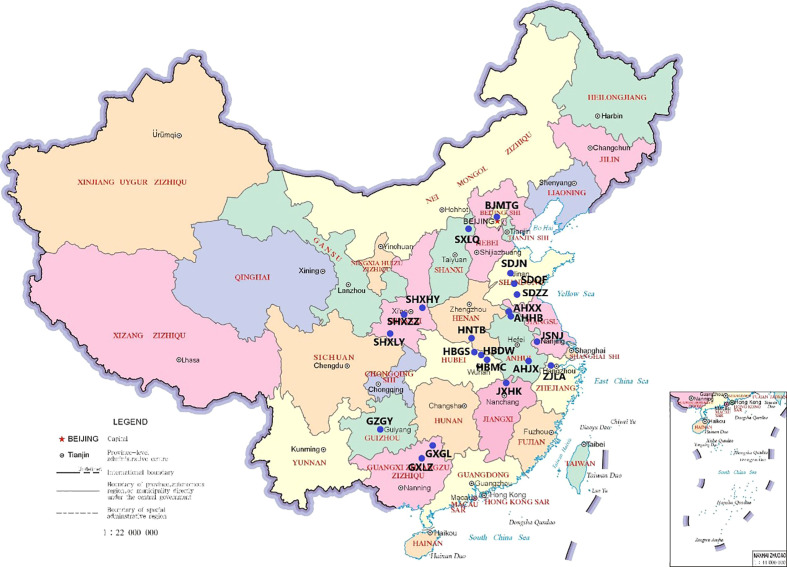
Sampling distribution diagram. Abbreviations are defined in **Table 1**.

Approximately a month before the ripening of *P. tatarinowii* fruit, when the fruit had just begun to turn from green to yellow (September to October 2022, **Figure 1**). A total of 21 natural populations (**Table 1**, **Figure 2**) were selected as sampling points through field investigation, and 10 pest- and disease-free adult plants of similar ages (with all trees being more than 100 years old) with normal growth were selected from each population. The plant spacing among the selected individuals was greater than 50 m (to avoid close relatives). More than 1 kg of leaves was collected from the east, south, west, and north directions of the middle and upper parts of a single tree crown and brought back to the laboratory. After drying naturally, individuals from the same population were mixed thoroughly mixed to form a sample.

**Table 1:** Location of the natural populations of *P. tatarinowii and* climatic conditions of the locations.

Order	Collection site	Code	Longitude (°E)	Latitude (°N)	Elevation (m)	Annual average temperature (°C)	Annual rainfall (mm)	Frost-free season (d)
1	Mentougou, Beijing	BJMTG	116.0207	40.04528	582.9	11.6	515.7	182
2	Lingqiu, Shanxi	SXLQ	114.0017	39.08971	763.0	7.0	433.3	145
3	Jinan, Shandong	SDJN	116.9816	36.36202	300.5	12.9	697.0	195
4	Qufu, Shandong	SDQF	117.1300	35.47904	152.5	13.6	701.1	199
5	Zaozhuang, Shandong	SDZZ	117.3354	34.46133	130.0	14.5	872.9	214
6	Tongbai, Henan	HNTB	113.3569	32.35258	264.0	15.0	1,168.0	231
7	Huayin, shaanxi	SHXHY	110.0827	34.52509	429.3	13.5	599.0	208
8	Zhouzhi, Shaanxi	SHXZZ	108.3297	34.05367	622.3	13.2	674.3	225
9	Lueyang, Shaanxi	SHXLY	106.1455	33.29699	715.8	13.2	860.0	236
10	Guangshui, Hubei	HBGS	113.9474	31.84771	228.9	14.0	990.0	221
11	Dawu, Hubei	HBDW	114.3340	31.71599	363.8	15.4	1,086.4	236
12	Macheng, Hubei	HBMC	115.3535	31.32564	347.3	16.8	1,291.5	246
13	Jingxian, Anhui	AHJX	118.3120	30.67469	89.4	15.7	1,500.0	239
14	Huaibei, Anhui	AHHB	116.9728	33.89653	212.5	14.7	849.6	203
15	Xiaoxian, Anhui	AHXX	117.0553	34.02167	177.5	14.5	814.0	205
16	Nanjing, Jiangsu	JSNJ	118.8175	32.14652	23.3	15.4	1,090.4	237
17	Linan, Zhejiang	ZJLA	119.4134	30.32065	400.1	16.4	1,613.9	237
18	Huko, Jiangxi	JXHK	116.2224	29.74495	45.0	17.4	1,442.5	259
19	Liuzhou, Guangxi	GXLZ	109.4524	24.31852	93.0	20.5	1,550.0	310
20	Guilin, Guangxi	GXGL	110.2815	25.26273	156.3	19.1	1,926.0	309
21	Guiyang, Guizhou	GZGY	106.7032	26.35821	1,048.5	15.3	1,129.5	270

#### Soil collection

When collecting soil samples, the surface humus was removed using tools. Five soil samples (0–20 cm) were collected from plants 1 m from the plant stem. The soil area of each soil sample was 20 cm × 20 cm, and stones and plant roots were removed. Soil samples were air-dried, ground, and passed through a 100-mesh sieve. After the soil samples from the same population were evenly mixed, 1 kg was used as a reserve for laboratory analysis.

GPS was used to observe the longitude, latitude, and altitude of the populations, and meteorological factors were obtained from the local meteorological department (**Table 1**).

### Test methods

#### Determination of leaf forage indices

Descriptions of the most used feed ingredients and general ingredients can be found in the Chinese feed database (http://www.chinafeeddata.org.cn). The determination indices included dry matter (DM) (direct drying method, GB/T 6435-2014), crude protein (CP) (Kjeldahl method, GB/T 6432-1994), crude fat (EE) (Soxhlet extraction method, GB/T 6433-1994), crude fiber (CF) (acid and alkali washing method, GB/T 6434-1994), ash (Ash) (550 °C burning, GB/T 6438-2007), neutral detergent fiber (NDF) (GB/T 20806-2006), and acid detergent fiber (ADF) (NY/T 1459-2007). The nitrogen-free extract (NEF) content was calculated as follows: NEF = DM − CP − CF − EE − ash. The content of calcium (Ca) in the leaves was determined using atomic absorption spectrometry (NY/T 1994-2010). Phosphorus (P) content was determined by spectrophotometry (GB/T 6437-2002). The tannin content in the leaves was determined using the phosphomolybdate sodium tungstate (F-D) colorimetric method (GB/T 27985—2011) (Robbins et al., 1987).

#### Calculation of leaf forage quality evaluation index

The relative feeding value (RFV) was calculated according to Rohweder et al. (1978) method: RFV = DMI (%DW) × DDM (%DW)/1.29, where DMI is dry matter intake (DMI = 120/NDF (unit is percentage of body weight, i.e., %DW)), and DDM is digestible dry matter (DDM = 88.9–0.779 ADF (unit is %DM)).

The *in vitro* dry matter digestibility (IVDMD) and metabolizable energy (ME) were calculated based on the prediction model of Li et al. (2012). IVDMD (%) = 246.083 − 0.676DM + 3.404EE − 0.739ADF + 0.120NDF − 1.734OM, where OM (organic matter) = DM-Ash; ME (MJ·kg^−1^) = 17.184 − 0.137DM − 0.203CP + 0.506EE − 0.185ADF + 0.067NDF.

#### Detection of amino acid content in leaves

Three populations were randomly selected as samples (SDJN, HBGS, and GXGL), and amino acids in the feed were detected according to GB/T 18246-2019. Free amino acids were determined as follows: 0.1 g of leaves were mixed with ultrapure water (1.5 ml) and then filtered through a 0.22 μm filter membrane after ultrasonication for 30 min. The results were determined by ultra-performance liquid chromatography in combination with a triple quadrupole mass spectrometer on a Hypersil Gold C18 (100 mm × 2.1 mm, 3.5 μm) column. Hydrolyzed amino acids were determined as follows: a 0.1 g sample of leaves was obtained and acidified with 6 mol·L^−1^ hydrochloric acid, and the amino acid contents were determined by the instrument described above.

#### Soil index determination

Soil pH value was measured using an SJ-4A-type pH meter. Available P, K, and Ca in the soil were determined using plasma ICP-AES after 1 mol·L^−1^ ammonium acetate extraction. The hydrolyzed N content was measured using the alkali diffusion method. The soil organic matter content was determined using organic matter photometry.

### Statistical methods

The data were subjected to analysis of variance and compared using Tukey’s honestly significant difference test using IBM SPSS software (version 26.0; IBM Corp., Armonk, NY, USA). Correlation analysis was performed by calculating the Spearman’s correlation coefficients. The Euclidean distance-leveling method was used for cluster analysis.

## Results and analysis

### Determination and evaluation of leaf nutrients in different populations

There were significant differences in the 11 leaf forage indices at the population level (**Table 2**). The dry matter content showed minimal differences, ranging from 94.23% to 95.29%, with an average of 94.79%. The crude protein content varied greatly among the populations, ranging from 10.77% to 18.65%, with an average value of 14.52%. The maximum value was observed in the SDJN population (18.65 ± 0.15%), and the minimum value was observed in the SHXLY population (10.77 ± 0.26%). Crude fat, crude fiber, and crude ash showed minimal differences among populations at 5.62%–7.62%, 5.26%–8.94%, and 15.54%–20.53%, respectively. The average nitrogen-free extract was 47.99%, the maximum value occurred in the SHXLY population (54.48 ± 0.59%), and the minimum value occurred in the SHXZZ population (43.48 ± 0.11%). The neutral detergent fiber content was significantly higher than that of acid detergent fiber (25.33% and 17.52%, respectively), and the two indices were significantly different between the populations (19.25%–33.20% and 14.34%–25.02%, respectively). The average calcium content in the leaves was 58.01 g·kg^−1^, the JXHK population had the highest calcium content (78.48 ± 0.08 g·kg^−1^), the HBDW population had a calcium content of (38.49 ± 0.70 g·kg^−1^), and the average phosphorus content in the leaves was only 0.785 g·kg^−1^, ranging from 0.521 g·kg^−1^ to 1.217 g·kg^−1^. The tannin content in the leaves was low, with an average of 4.97 g·kg^−1^, and the highest tannin content was only 5.59 g·kg^−1^ in the SHXLY population, while the lowest was 4.45 g·kg^−1^ in the GXLZ population.

**Table 2:** Statistical analysis of leaf forage indices for the different populations of *P. tatarinowii*.

Populations	Leaf forage indices										
	DM (%)	CP (%)	EE (%)	CF (%)	Ash (%)	NEF (%)	NDF (%)	ADF (%)	P (g·kg^−1^)	Ca (g·kg^−1^)	Tannins (g·kg^−1^)
BJMTG	94.58 ± 0.02^IJ^	15.2 ± 0.31^G^	6.67 ± 0.32^DE^	8.94 ± 0.05^A^	16.87 ± 0.30^EFGH^	46.89 ± 0.41^FGH^	26.50 ± 0.46^F^	16.69 ± 0.24^FG^	0.728 ± 0.002^J^	60.18 ± 0.20^F^	4.58 ± 0.01^M^
SXLQ	94.85 ± 0.07^D^	16.30 ± 0.26^CD^	6.67 ± 0.16^DE^	6.08 ± 0.07^N^	16.12 ± 0.99^GHI^	49.68 ± 1.55^DE^	19.56 ± 0.31^LM^	15.17 ± 0.61^IJ^	0.562 ± 0.001^P^	51.39 ± 0.27^MN^	4.96 ± 0.02^FGH^
SDJN	94.69 ± 0.02^GH^	18.65 ± 0.15^A^	6.26 ± 0.22^FGH^	8.19 ± 0.07^DE^	17.40 ± 0.45^DEF^	44.18 ± 0.76^IJ^	31.31 ± 0.45^B^	18.51 ± 0.50^DE^	0.736 ± 0.001^I^	52.86 ± 0.13^KL^	4.80 ± 0.02^JK^
SDQF	94.74 ± 0.01^FG^	11.53 ± 0.31^L^	6.11 ± 0.05^GH^	8.13 ± 0.08^EFG^	17.13 ± 0.21^DEFG^	51.85 ± 0.63^BC^	24.32 ± 0.44^HIJ^	15.99 ± 0.45^GHI^	0.712 ± 0.003^L^	46.98 ± 0.28°	5.14 ± 0.03^CD^
SDZZ	95.17 ± 0.06^B^	14.39 ± 0.08^H^	6.50 ± 0.28^EFG^	7.34 ± 0.11^L^	15.9 ± 0.55^HI^	51.01 ± 0.25^CD^	19.73 ± 0.28^LM^	14.34 ± 0.51^J^	0.606 ± 0.004°	61.55 ± 0.33^E^	5.08 ± 0.02^DE^
HNTB	95.21 ± 0.01^B^	14.66 ± 0.23^H^	7.00 ± 0.23^CD^	7.87 ± 0.11^HIJ^	16.81 ± 0.22^EFGH^	48.86 ± 0.30^E^	30.29 ± 0.34^BCD^	19.16 ± 0.60^CD^	1.217 ± 0.005^A^	69.87 ± 0.50^C^	4.93 ± 0.07^GHI^
SHXHY	95.05 ± 0.06^C^	12.07 ± 0.23^JK^	7.20 ± 0.12^BC^	8.16 ± 0.15^EF^	20.53 ± 0.49^A^	47.09 ± 1.04^FG^	20.57 ± 0.68^KL^	15.95 ± 0.84^GHI^	0.698 ± 0.001^M^	65.14 ± 2.04^D^	5.02 ± 0.09^EFG^
SHXZZ	94.38 ± 0.03^L^	16.06 ± 0.16^DE^	7.62 ± 0.11^A^	8.33 ± 0.02^D^	18.88 ± 0.10^BC^	43.48 ± 0.11^J^	25.85 ± 0.17^FG^	17.22 ± 0.66^F^	0.933 ± 0.003^E^	57.14 ± 0.70^I^	4.78 ± 0.03^JK^
SHXLY	95.29 ± 0.02^A^	10.77 ± 0.26^M^	6.73 ± 0.05^DE^	5.26 ± 0.04°	18.07 ± 0.82^CD^	54.48 ± 0.59^A^	19.60 ± 0.49^LM^	19.31 ± 0.66^CD^	0.750 ± 0.001^H^	55.00 ± 0.23^J^	5.59 ± 0.03^A^
HBGS	94.23 ± 0.03^M^	14.56 ± 0.16^H^	5.62 ± 0.16^I^	7.89 ± 0.11^HI^	16.49 ± 0.45^FGHI^	49.68 ± 0.59^DE^	33.20 ± 0.78^A^	25.02 ± 0.50^A^	0.874 ± 0.003^F^	57.52 ± 0.27^HI^	5.55 ± 0.05^A^
HBDW	94.57 ± 0.02^IJ^	15.22 ± 0.10^G^	7.03 ± 0.33^CD^	7.72 ± 0.02^J^	17.76 ± 0.22^DE^	46.85 ± 0.69^GH^	29.09 ± 0.55^E^	19.75 ± 0.50^C^	0.794 ± 0.001^G^	38.49 ± 0.70^P^	4.87 ± 0.10^HIJ^
HBMC	94.53 ± 0.02^JK^	14.46 ± 0.12^H^	7.54 ± 0.11^AB^	7.52 ± 0.03^K^	19.54 ± 0.51^AB^	45.46 ± 0.78^GHI^	29.50 ± 0.49^DE^	22.98 ± 0.77^B^	0.721 ± 0.001^K^	50.23 ± 0.23^N^	4.87 ± 0.03^HIJ^
AHJX	94.78 ± 0.02^DEF^	15.29 ± 0.23^FG^	6.88 ± 0.12^CDE^	6.15 ± 0.01^N^	17.70 ± 0.28^DE^	48.76 ± 0.63^E^	21.43 ± 0.45^K^	16.45 ± 0.51^FGH^	0.798 ± 0.004^G^	52.30 ± 0.50^LM^	5.07 ± 0.04^DEF^
AHHB	94.77 ± 0.05^DEF^	15.76 ± 0.21^E^	6.66 ± 0.25^DE^	8.02 ± 0.10^FGH^	19.24 ± 0.69^B^	45.09 ± 1.29^IJ^	25.35 ± 0.73^GH^	15.93 ± 0.71^GHI^	1.193 ± 0.003^B^	72.99 ± 0.27^B^	4.64 ± 0.03^LM^
AHXX	95.19 ± 0.01^B^	17.46 ± 0.10^B^	6.00 ± 0.23^HI^	7.97 ± 0.05^GH^	19.24 ± 0.66^B^	44.52 ± 0.95^IJ^	23.52 ± 0.49^J^	15.33 ± 0.52^HIJ^	0.792 ± 0.004^G^	58.52 ± 0.28^GH^	4.83 ± 0.11^IJ^
JSNJ	94.75 ± 0.04^EFG^	11.84 ± 0.08^KL^	6.75 ± 0.06^DE^	7.75 ± 0.03^IJ^	15.54 ± 0.27^I^	52.88 ± 0.14^AB^	23.52 ± 0.47^J^	14.63 ± 0.37^J^	0.966 ± 0.004^D^	59.77 ± 0.91^FG^	5.30 ± 0.03^B^
ZJLA	94.83 ± 0.06^DE^	12.31 ± 0.02^J^	7.66 ± 0.05^A^	8.76 ± 0.04^B^	19.09 ± 0.16^B^	47.01 ± 0.20^FGH^	19.25 ± 0.58^M^	16.62 ± 0.38^FG^	0.521 ± 0.001^R^	62.08 ± 1.33^E^	5.22 ± 0.02^BC^
JXHK	94.68 ± 0.02^GH^	15.69 ± 0.26^EF^	7.52 ± 0.22^AB^	7.24 ± 0.04^L^	18.83 ± 0.17^BC^	45.40 ± 0.71^HI^	30.95 ± 0.28^BC^	16.48 ± 0.40^FG^	1.167 ± 0.002^C^	78.48 ± 0.08^A^	4.94 ± 0.06^GHI^
GXLZ	94.64 ± 0.03^HI^	16.69 ± 0.25^C^	6.65 ± 0.22^EF^	8.59 ± 0.04^C^	15.71 ± 0.33^I^	47.00 ± 0.86^FGH^	24.64 ± 0.39^HI^	17.49 ± 0.41^EF^	0.671 ± 0.002^N^	56.59 ± 0.20^I^	4.45 ± 0.01^N^
GXGL	95.20 ± 0.06^B^	12.87 ± 0.15^I^	7.43 ± 0.29^AB^	7.05 ± 0.09^M^	19.29 ± 0.71^B^	48.55 ± 1.29^EF^	30.22 ± 0.77^CD^	17.31 ± 0.66^F^	0.525 ± 0.002^QR^	54.03 ± 0.23^JK^	4.96 ± 0.02^FGH^
GZGY	94.48 ± 0.02^K^	13.18 ± 0.17^I^	6.13 ± 0.06^GH^	7.03 ± 0.15^M^	19.10 ± 0.70^BC^	49.03 ± 1.11^E^	23.63 ± 0.17^IJ^	17.53 ± 0.46^EF^	0.528 ± 0.003^Q^	57.10 ± 0.20^I^	4.71 ± 0.10^KL^
Average	94.79 ± 0.29	14.52 ± 2.05	6.79 ± 0.59	7.62 ± 0.90	17.87 ± 1.49	47.99 ± 2.96	25.33 ± 4.39	17.52 ± 2.62	0.785 ± 0.207	58.01 ± 8.72	4.97 ± 0.29
F	182.427**	326.829**	25.637**	412.427**	25.130**	39.455**	240.179**	67.101**	17,371.365**	547.822**	88.298**

**Significant at p <0.01. Different capital letters in the same column indicate significant differences (P <0.01).

### Analysis of leaf forage quality evaluation indices of different populations

The five forage quality evaluation indices showed highly significant differences among populations (**Table 3**). The average DMI was 4.88%; the highest DMI values were in the ZJLA (6.24 ± 0.19%), SXLQ (6.14 ± 0.10%), SHXLY (6.12 ± 0.15%), and SDZZ (6.08 ± 0.09%) populations, and the lowest value was in the HBGS population (3.62 ± 0.09%). The average DDM was 75.25%, the highest value was in the SDZZ population (77.73 ± 0.40%), and the lowest was in the HBGS population (65.05 ± 0.39%). The difference in RFV was high, ranging from 194.57% to 367.29%, with an average of 285.43%. In comparison to the other populations, the ZJLA (367.29 ± 12.34%), SXLQ (366.71 ± 8.16%), and SDZZ (366.48 ± 6.94%) population had higher RFV values, and the HBGS population (194.57 ± 3.51%) had the lowest value. The average predicted value of IVDMD was 61.84%, with the highest values occurring in the SHXHY (67.82 ± 0.87%), SHXZZ (67.68 ± 0.16%), and JXHK (67.68 ± 0.16%) populations and the lowest value occurring in the HBGS population (52.20 ± 1.88%). The average predicted value of ME was 3.14 MJ·kg^−1^, ranging from 1.76 MJ·kg^−1^ (HBGS) to 4.11 MJ·kg^−1^ (GXGL).

**Table 3:** The difference in the forage quality evaluation indices among the different populations of *P. tatarinowii*.

Populations	CP (%)	NDF (%)	ADF (%)	DMI (%)	DDM (%)	RFV (%)	IVDMD (%)	ME (MJ·kg^-1^)
BJMTG	15.2 ± 0.31^G^ Level 2	26.50 ± 0.46^F^	16.69 ± 0.24^FG^	4.53 ± 0.08^G^	75.90 ± 0.19^DEF^	266.44 ± 4.07^G^	60.96 ± 0.80^EFGH^	3.20 ± 0.15^DE^
SXLQ	16.30 ± 0.26^CD^ Level 2	19.56 ± 0.31^LM^	15.17 ± 0.61^IJ^	6.14 ± 0.10^A^	77.08 ± 0.47^ABC^	366.71 ± 8.16^A^	59.30 ± 2.04^EFGH^	2.76 ± 0.05^HI^
SDJN	18.65 ± 0.15^A^ Level 1	31.31 ± 0.45^B^	18.51 ± 0.50^DE^	3.83 ± 0.05^IJ^	74.48 ± 0.39^GH^	221.36 ± 4.30^H^	59.44 ± 1.23^EFGH^	2.26 ± 0.02^J^
SDQF	11.53 ± 0.31^L^ Level 3	24.32 ± 0.44^HIJ^	15.99 ± 0.45^GHI^	4.94 ± 0.09^DE^	76.45 ± 0.35^BCDE^	292.45 ± 3.94^DE^	59.37 ± 0.90^EFGH^	3.63 ± 0.08^C^
SDZZ	14.39 ± 0.08^H^ Level 2	19.73 ± 0.28^LM^	14.34 ± 0.51^J^	6.08 ± 0.09^A^	77.73 ± 0.40^A^	366.48 ± 6.94^A^	58.25 ± 0.29^H^	3.18 ± 0.06^EF^
HNTB	14.66 ± 0.23^H^ Level 2	30.29 ± 0.34^BCD^	19.16 ± 0.60^CD^	3.96 ± 0.04^HI^	73.98 ± 0.47^H^	227.20 ± 3.94^H^	59.09 ± 0.70^FGH^	3.19 ± 0.07^F^
SHXHY	12.07 ± 0.23^JK^ Level 3	20.57 ± 0.68^KL^	15.95 ± 0.84^GHI^	5.84 ± 0.19^B^	76.48 ± 0.65^BCDE^	346.18 ± 14.36^BC^	67.82 ± 0.87^A^	3.78 ± 0.09^BC^
SHXZZ	16.06 ± 0.16^DE^ Level 2	25.85 ± 0.17^FG^	17.22 ± 0.66^F^	4.64 ± 0.03^FG^	75.49 ± 0.52^EFG^	271.68 ± 3.61^FG^	67.68 ± 0.16^A^	3.40 ± 0.02^D^
SHXLY	10.77 ± 0.26^M^ Level 4	19.60 ± 0.49^LM^	19.31 ± 0.66^CD^	6.12 ± 0.15^A^	73.86 ± 0.52^H^	350.71 ± 11.23^AB^	58.73 ± 1.18^GH^	3.09 ± 0.02^EF^
HBGS	14.56 ± 0.16^H^ Level 2	33.20 ± 0.78^A^	25.02 ± 0.50^A^	3.62 ± 0.09^J^	69.41 ± 0.39^J^	194.57 ± 3.51^I^	52.20 ± 1.88^I^	1.76 ± 0.26^K^
HBDW	15.22 ± 0.10^G^ Level 2	29.09 ± 0.55^E^	19.75 ± 0.50^C^	4.13 ± 0.08^H^	73.52 ± 0.39^H^	235.19 ± 5.65^H^	61.77 ± 1.28^DEFG^	2.99 ± 0.10^EFG^
HBMC	14.46 ± 0.12^H^ Level 2	29.50 ± 0.49^DE^	22.98 ± 0.77^B^	4.07 ± 0.07^HI^	71.00 ± 0.60^I^	223.95 ± 5.61^H^	64.38 ± 1.78^BCD^	2.84 ± 0.14^GH^
AHJX	15.29 ± 0.23^FG^ Level 2	21.43 ± 0.45^K^	16.45 ± 0.51^FGH^	5.60 ± 0.12^C^	76.08 ± 0.40^CDEF^	330.38 ± 8.57^C^	62.20 ± 0.61^CDE^	2.97 ± 0.05^FG^
AHHB	15.76 ± 0.21^E^ Level 2	25.35 ± 0.73^GH^	15.93 ± 0.71^GHI^	4.74 ± 0.14^EFG^	76.49 ± 0.55^BCDE^	280.91 ± 10.06^EFG^	64.98 ± 1.70^ABC^	3.12 ± 0.01^EF^
AHXX	17.46 ± 0.10^B^ Level 1	23.52 ± 0.49^J^	15.33 ± 0.52^HIJ^	5.10 ± 0.11^D^	76.96 ± 0.41^ABCD^	304.50 ± 7.90^D^	61.96 ± 1.64^DEF^	2.37 ± 0.05^J^
JSNJ	11.84 ± 0.08^KL^ Level 3	23.52 ± 0.47^J^	14.63 ± 0.37^J^	5.10 ± 0.10^D^	77.50 ± 0.29^AB^	306.58 ± 7.30^D^	59.65 ± 0.56^EFGH^	4.08 ± 0.03^A^
ZJLA	12.31 ± 0.02^J^ Level 3	19.25 ± 0.58^M^	16.62 ± 0.38^FG^	6.24 ± 0.19^A^	75.95 ± 0.30^DEF^	367.29 ± 12.34^A^	66.75 ± 0.07^AB^	3.78 ± 0.02^BC^
JXHK	15.69 ± 0.26^EF^ Level 2	30.95 ± 0.28^BC^	16.48 ± 0.40^FG^	3.88 ± 0.04^I^	76.06 ± 0.31^CDEF^	228.61 ± 3.01^H^	67.68 ± 0.83^A^	3.86 ± 0.01^B^
GXLZ	16.69 ± 0.25^C^ Level 2	24.64 ± 0.39^HI^	17.49 ± 0.41^EF^	4.87 ± 0.08^DEF^	75.28 ± 0.32^FG^	284.28 ± 5.65^EF^	57.93 ± 1.13^H^	2.61 ± 0.02^I^
GXGL	12.87 ± 0.15^I^ Level 3	30.22 ± 0.77^CD^	17.31 ± 0.66^F^	3.97 ± 0.10^HI^	75.42 ± 0.51^EFG^	232.27 ± 7.52^H^	66.25 ± 1.98^AB^	4.11 ± 0.06^A^
GZGY	13.18 ± 0.17^I^ Level 3	23.63 ± 0.17^IJ^	17.53 ± 0.46^EF^	5.08 ± 0.04^D^	75.24 ± 0.36^FG^	296.21 ± 3.51^DE^	62.26 ± 1.14^CDE^	3.01 ± 0.08^EFG^
Average	14.52 ± 2.05	25.33 ± 4.39	17.52 ± 2.62	4.88 ± 0.85	75.25 ± 2.04	285.43 ± 54.01	61.84 ± 4.10	3.14 ± 0.60
F	326.829**	240.179**	67.101**	206.306**	67.215**	162.401**	32.425**	140.106**

Different capital letters in the same column indicate significant differences (P <0.01). **Significant at p <0.01.

The nutritional quality of the samples was assessed using the American Forage and Grassland Association (AFGA) hay grade standard (**Supplementary Table 1**) (Li et al., 2020). The results (**Table 3**) show that the NDF (all less than 40), ADF (all less than 31), DMI (all more than 3.0), DDM (all more than 65), and RFV (all more than 151) of the tested samples reached the super standard. Only the crude protein index showed a significant grade difference. Among the 21 samples, two met the grade 1 standard, namely the SDJN and AHXX populations, 12 met the grade 2 standard, six met the grade 3 standard, and one met the grade 4 standard. In general, the crude protein content of 2/3 of the samples was more than 14%, which met or exceeded the grade 2 standard, and the other indices met the super standards, indicating that the forage characteristics of the *P. tatarinowii* leaves were excellent. Therefore, this species is a high-quality raw forage material. Based on the above forage evaluation indices, we concluded that the leaf forage characteristics of the SXLQ, SDJN, SDZZ, and AHXX populations were the best.

### Differences in amino acid content

Proteins are composed of amino acids that are essential nutrients. Therefore, the ratio of amino acids in feed is key to evaluating the nutritional value of protein in feed. Taking SDJN, HBGS, and GXGL as test materials (**Supplementary Table 2**), the average total amount (T) of hydrolyzed amino acids detected was 108.20 mg·g^−1^, with significant differences among samples. The content of SDJN (124.71 mg·g^−1^) was higher than that of HBGS (110.17 mg·g^−1^) and GXGL (89.73 mg·g^−1^). The hydrolyzed amino acids contain 19 kinds of amino acids, of which LEU (32.281 mg·g^−1^) had the highest content, followed by isoleucine (ILE) (25.99 mg·g^−1^) and tyrosine (TYR) (16.11 mg·g^−1^). Proline (PRO) (4.25 mg·g^−1^), phenylalanine (PHE) (3.55 mg·g^−1^) and serine (SER) (3.49 mg·g^−1^) also accounted for a certain proportion of the total; the content of the remaining amino acids were low, generally 1 mg·g^−1^.

The average total amount of free amino acids was 47.87 mg·g^−1^, and the difference between the samples was very significant. The content of HBGS (50.56 mg·g^−1^) was higher than that of SDJN (47.75 mg·g^−1^) and GXGL (45.30 mg·g^−1^). There were 18 kinds of free amino acids, among which isoleucine (ILE) had the highest content (22.89 ± 3.05 mg·g^−1^), followed by leucine (LEU) (10.86 ± 1.62 mg·g^−1^), threonine (THR) (5.28 ± 0.81 mg·g^−1^), and serine (SER) (2.47 ± 0.52 mg·g^−1^). The contents of the other amino acids were below 1 mg·g^−1^.

### Analysis of soil physical and chemical properties of the different populations

The six soil physicochemical property indices showed highly significant differences among populations (**Table 4**). The pH value ranged from 6.22 to 7.43, with an average value of 7.01, and the difference in pH values between the north and south was significant. There were 13 populations with a pH value greater than or equal to 7 (mostly the northern populations), and 8 populations had a pH value less than 7 (mostly the southern populations). The soil available P, K, and Ca contents varied greatly among the populations, and the K content was the highest, followed by P and Ca, with K, P, and Ca contents ranging from 44.93 mg·kg^−1^ (SDJN) to 299.40 mg·kg^−1^ (HNTB), 12.29 mg·kg^−1^ (SDQF) to 77.26 mg·kg^−1^ (GXLZ), and 3.46 mg·kg^−1^ (ZJLA) to 29.33 mg·kg^−1^ (SDQF), respectively. The content of hydrolyzed N in the soil varied greatly, with the highest content occurring in the GXLZ population and reaching 293.99 mg·kg^−1^, while the lowest content occurred in the AHHB population and was only 55.94 mg·kg^−1^. Similarly, the soil organic matter content was highest in the GXLZ population (23.91 ± 0.09%) and lowest in the AHHB population (3.39 ± 0.24%).

**Table 4:** Statistical analysis of soil physicochemical property indices of the different populations.

	Soil physicochemical property indices					
Population	pH	P (mg·kg^−1^)	K (mg·kg^−1^)	Ca (mg·kg^−1^)	N (mg·kg^−1^)	soil organic matter (%)
BJMTG	7.43 ± 0.01^A^	19.15 ± 0.03^G^	165.08 ± 1.42^F^	14.71 ± 0.63^DE^	156.30 ± 4.06^FG^	11.75 ± 0.20^E^
SXLQ	7.24 ± 0.02^C^	15.07 ± 0.11^N^	113.17 ± 2.45^K^	9.45 ± 0.61^HI^	182.53 ± 5.73^E^	12.42 ± 0.07^D^
SDJN	7.15 ± 0.01^E^	23.17 ± 0.03^I^	44.93 ± 2.84°	11.36 ± 0.85^GH^	146.30 ± 7.74^GH^	10.80 ± 0.08^G^
SDQF	7.32 ± 0.02^B^	12.29 ± 0.12^Q^	167.50 ± 1.08^F^	29.33 ± 1.66^A^	74.69 ± 4.82^K^	5.61 ± 0.08^M^
SDZZ	6.97 ± 0.02^GH^	23.41 ± 0.37^I^	97.97 ± 0.54^M^	15.88 ± 1.02^DE^	178.29 ± 4.60^E^	12.65 ± 0.03^D^
HNTB	7.29 ± 0.01^B^	27.30 ± 0.03^H^	299.40 ± 1.43^A^	5.21 ± 0.82^KL^	143.99 ± 5.52^H^	13.27 ± 0.10^C^
SHXHY	7.00 ± 0.02^G^	43.23 ± 0.02^D^	194.59 ± 5.69^D^	13.84 ± 1.00^EF^	104.95 ± 2.63^I^	9.87 ± 0.10^H^
SHXZZ	6.22 ± 0.02^N^	17.77 ± 0.05^H^	137.35 ± 1.94^I^	6.09 ± 0.15^JK^	61.58 ± 7.20^L^	6.47 ± 0.11^L^
SHXLY	7.20 ± 0.02^CD^	33.06 ± 0.05^G^	160.57 ± 1.94^G^	27.90 ± 1.45^AB^	165.24 ± 5.10^F^	15.58 ± 0.20^B^
HBGS	7.09 ± 0.01^F^	17.09 ± 0.04^K^	99.51 ± 1.86^LM^	4.24 ± 0.87^KL^	59.86 ± 6.64^L^	6.35 ± 0.17^L^
HBDW	6.83 ± 0.02^K^	14.60 ± 0.12°	78.54 ± 1.03^N^	5.60 ± 0.69^JKL^	105.30 ± 4.05^I^	7.52 ± 0.12^J^
HBMC	7.32 ± 0.01^B^	42.81 ± 0.05^D^	226.40 ± 1.87^B^	7.70 ± 0.96^IJ^	146.69 ± 4.04^GH^	12.50 ± 0.05^D^
AHJX	6.64 ± 0.04^L^	39.87 ± 0.07^E^	133.58 ± 1.61^I^	4.96 ± 0.21^KL^	216.52 ± 5.30^C^	9.68 ± 0.10^HI^
AHHB	6.61 ± 0.02^L^	15.66 ± 0.02^M^	183.67 ± 0.93^E^	6.31 ± 1.81^JK^	55.94 ± 3.44^L^	3.39 ± 0.24^N^
AHXX	6.51 ± 0.02^M^	13.89 ± 0.03^P^	103.64 ± 0.93^L^	9.88 ± 0.82^GHI^	86.49 ± 3.64^J^	7.09 ± 0.19^K^
JSNJ	7.10 ± 0.02^F^	57.50 ± 0.07^B^	167.17 ± 1.94^F^	11.86 ± 0.61^FG^	148.09 ± 3.96^GH^	9.68 ± 0.11^HI^
ZJLA	6.95 ± 0.01^HI^	16.57 ± 0.02^L^	112.13 ± 0.54^K^	3.46 ± 0.63^L^	178.79 ± 6.65^E^	11.44 ± 0.06^F^
JXHK	6.92 ± 0.02I^G^	56.13 ± 0.02^C^	129.08 ± 0.54^J^	19.98 ± 0.77^C^	106.58 ± 3.15^I^	9.42 ± 0.07^I^
GXLZ	6.90 ± 0.02^G^	77.26 ± 0.78^A^	126.72 ± 1.07^J^	26.49 ± 1.16^B^	293.99 ± 4.06^A^	23.91 ± 0.09^A^
GXGL	7.30 ± 0.01^B^	17.78 ± 0.10^K^	146.02 ± 1.42^H^	16.76 ± 0.99^D^	230.85 ± 2.98^B^	13.11 ± 0.06^C^
GZGY	7.20 ± 0.02^D^	36.11 ± 0.32^F^	214.50 ± 0.93^C^	16.85 ± 1.27^D^	196.69 ± 2.52^D^	12.42 ± 0.16^D^
Average	7.01 ± 0.31	29.55 ± 17.37	147.69 ± 55.69	12.75 ± 7.83	144.75 ± 61.06	10.71 ± 4.23
F	1,041.175**	20,923.545**	2,516.160**	192.624**	483.298**	3,454.039**

**Significant at p <0.01. Different capital letters in the same column indicate significant differences (P <0.01).

### Correlation analysis between forage indices and soil physicochemical properties, geography, and ecological factors

According to the correlation analysis between forage indices and geographic and ecological factors (**Table 5**), EE had a significant or extremely significant positive correlation with annual mean temperature (0.286*), annual mean precipitation (0.396**), and frost-free period (0.296*). The results showed that increasing the temperature and water content increased the crude fat content of *P. tatarinowii* leaves. There was a significant positive correlation between CF and longitude (0.282*), but the crude fiber content in the forage was not too high; thus, the central and western regions of the distribution area could be selected as the planting area. NDF was negatively correlated with elevation (−0.278*) and positively correlated with average annual temperature (0.285*). The lower the NDF content, the higher the forage value. Therefore, planting in a slightly higher elevation area should occur. The DMI was positively correlated with elevation (0.283*) and negatively correlated with the average annual temperature (−0.313*) and frost-free period (−0.258*), while the RFV was negatively correlated with the average annual temperature (−0.312*) and frost-free period (−0.271*). The results showed that appropriately increasing the elevation of the planting area and lowering the temperature of the planting area could improve leaf forage characteristics. There was a significant positive correlation between ME and annual precipitation (0.273*), indicating that increasing water content during cultivation improved leaf feeding characteristics. In general, *P. tatarinowii* can be planted to provide leaf forage in cold and cool areas at specific elevations, and the amount of irrigation can be increased.

**Table 5:** Correlation analysis of the forage indices with geographical and ecological factors, and soil physicochemical properties.

	Geographical and ecological factors						Soil physicochemical properties					
	Longitude	Latitude	Elevation	Annual average temperature	Annual rainfall	Frost-free season	PH	P	K	Ca	N	soil organic matter
DM	−0.024	0.046	−0.153	0.006	0.064	−0.007	0.114	−0.082	0.169	0.233	0.222	0.227
CP	0.202	0.166	−0.132	−0.097	−0.153	−0.199	−0.395^**^	−0.054	−0.409^**^	−0.331^**^	−0.036	−0.024
EE	−0.063	−0.202	−0.023	0.286^*^	0.396^**^	0.296^*^	−0.137	0.184	0.206	−0.173	0.148	0.130
CF	0.282^*^	0.070	−0.247	0.201	−0.073	−0.030	−0.124	−0.048	−0.029	−0.184	−0.249^*^	−0.144
Ash	−0.214	−0.174	0.212	0.177	0.139	0.152	−0.252^*^	−0.147	0.197	−0.151	−0.260^*^	−0.298^*^
NEF	−0.108	−0.004	0.049	−0.139	−0.014	0.011	0.476^**^	0.082	0.168	0.419^**^	0.224	0.207
NDF	0.023	−0.180	−0.278^*^	0.285^*^	0.214	0.238	0.158	−0.029	0.020	−0.184	−0.262^*^	−0.171
ADF	−0.231	−0.190	0.094	0.144	0.159	0.194	0.232	−0.034	0.084	−0.226	−0.149	0.025
P	0.192	0.103	−0.375^**^	0.064	−0.066	−0.077	−.303^*^	0.127	0.312^*^	−0.233	−0.534^**^	−0.389^**^
Ca	0.094	−0.039	−0.195	0.139	0.070	0.033	−0.139	0.249^*^	0.334^**^	−0.044	−0.167	-0.057
Tannins	0.072	0.073	−0.080	−0.159	0.025	−0.098	0.188	−0.128	−0.090	0.011	−0.191	-0.178
DMI	−0.015	0.192	0.283*	−0.313*	−0.201	−0.258*	−0.118	0.003	−0.063	0.156	0.265*	0.184
DDM	0.231	0.191	−0.094	−0.144	−0.159	−0.195	−0.232	0.034	−0.084	0.226	0.149	-0.026
RFV	0.026	0.206	0.242	−0.312*	−0.209	−0.271*	−0.137	0.002	−0.074	0.163	0.260*	0.161
IVDMD	−0.049	−0.149	0.069	0.218	0.221	0.180	−0.334**	0.023	0.158	−0.137	−0.093	-0.198
ME	0.029	−0.152	−0.122	0.231	0.273*	0.244	0.088	0.147	0.316*	0.220	0.075	-0.039

*At a level of 0.05 (two-tailed), the correlation was significant; **At a level of 0.01 (two-tailed), the correlation was significant.

Correlation analysis between forage indices and soil physical and chemical properties (**Table 5**) showed that CP had extremely significant or significantly negative correlations with K (−0.409**) and Ca (−0.331*) in the soil. Therefore, improving the crude protein content in leaves could be regulated by moderately reducing the K and Ca contents in the soil. CF, Ash, and NDF were significantly negatively correlated with soil nitrogen content (−0.249*, −0.260*, and −0.262*, respectively), ash was significantly negatively correlated with soil organic matter (−0.298*), and DMI and RFV were significantly positively correlated with N content (0.265* and 0.260*, respectively). These results indicate that increasing the soil N content and soil organic matter content could improve the forage value of *P. tatarinowii* leaves. Therefore, increasing nitrogen fertilizer and decreasing K and Ca fertilizers can further improve the leaf forage value of *P. tatarinowii* leaves when leaf forage value is the objective of cultivation.

### Hierarchical cluster analysis

**Figure 3:**
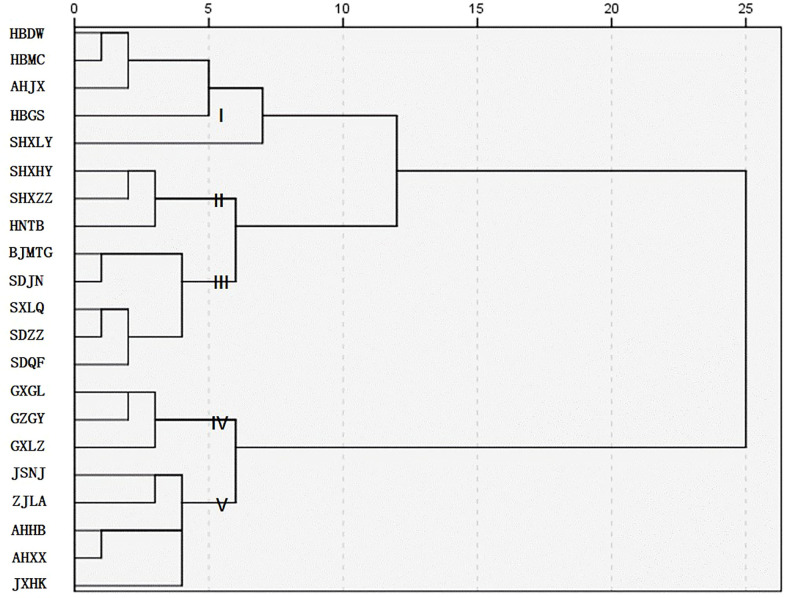
Cluster analysis of the *P. tatarinowii* forage indices.

**Figure 4:**
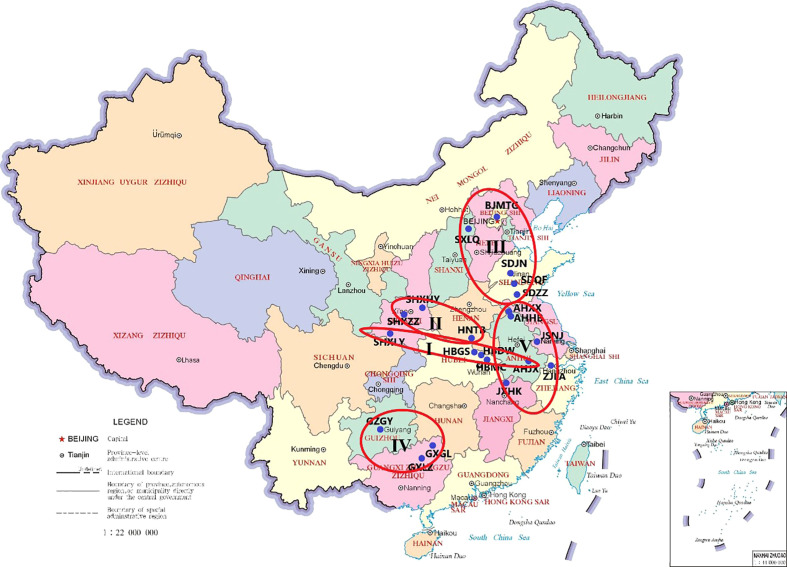
Species cluster zoning map of the *P. tatarinowii* forage indices.

The results of cluster analysis (**Figure 3**) showed that, when the distance was 5, group I included three populations in Hubei Province (HBDW, HBMC, and HBGS), one population in Anhui Province (AHJX), and one population in Shaanxi Province (SHXLY), mainly in the Yangtze River Basin; group II included two populations in Shaanxi Province (SHXHY and SHXZZ) and one population in Henan Province (HNTB); group III included five populations in Shandong Province, Beijing Province, and Shanxi Province (SDJN, SDQF, SDZZ, SXLQ, and BJMTG); group IV included two populations in Guangxi Province (GXGL and GXLZ) and one in Guizhou Province (GZGY), and group V included five populations in the eastern coastal areas of Anhui, Jiangsu, Jiangxi and Zhejiang Provinces (AHHB, AHXX, JSNJ, JXHK, and ZJLA). When the distance was 10, it was divided into three groups: groups II and III, which could be collectively referred to as the northern population, and groups IV and V, which were mainly the eastern coastal population and the southwest population. When the distance was 15, it was divided into two groups: the northern population and the Yangtze River Basin were grouped into one group including groups I, II, and III, and the eastern coastal areas and southwestern areas were grouped into one group including groups IV and V. The results of the cluster analysis were labeled in the sampling map (**Figure 4**), which showed that the division had obvious regional characteristics. Except for the overlapping part in Jingxian County, Anhui Province (AHJX), the distribution of the other groups was highly consistent with the geographical division. In general, the forage characteristics of *P. tatarinowii* leaves had obvious regional characteristics and were divided into four groups: the Yangtze River Basin population (I), northern population (II and III), southwestern population (IV), and eastern coastal population (V).

## Discussion

### Forage value evaluation of *P. tatarinowii* leaves

To fully analyze the forage characteristics of *P. tatarinowii* leaves, the results of this experiment were compared with those of the main woody and herbaceous diets reported in China and globally. Compared with other woody diets, the crude protein content of *P. tatarinowii* leaves (14.52%) was moderate. This result was similar to that of *Broussonetia papyrifera* (16.15%) (Shi et al., 2019), *R. pseudoacacia* (16.24%) (Li et al., 2006), *Lespedeza inschanica* (17.5%) (Wang et al., 2012), and *Folium mori* (17.86%) (Zeng et al., 2021); it was lower than that of *Moringa oleifera* (26.64%) (Zeng et al., 2021), *Lespedeza bicolor* (23.22%) (Wang et al., 2012), *L. daurica* (21.8%) (Wang et al., 2012), *L. leucocephala* (21.83%) (Li et al., 2012; Li et al., 2020), *A. fruticose* (21%) (Papachristou and Papanastasis, 1994), *Paulownia fortune* (20.28%) (Wu et al., 2017), and *Cnidoscolus aconitifolius* (19.2%) (Gu et al., 2021); and it was higher than that of *Neolamarckia cadamba* (13.36%) (Zeng et al., 2021), *Eucommia ulmoides* (12.22%) (Wang et al., 2017), and *Populus* (9.45%) (Wu et al., 2017). The crude protein content of *P. tatarinowii* leaves (14.52%) was higher than that of conventional herbaceous diets and was comparable to that of wheat bran or grade 2 alfalfa; for example, oat grass, rye, wheat bran, corn, grade 1 alfalfa, and grade 2 alfalfa had crude protein contents of 9.38%, 9.5%, 15.7%, 9.4%, 19.1%, and 17.2%, respectively in the China feed database (http://www.chinafeeddata.org.cn).

Studies have shown that the proper addition of crude fiber to feed can reduce the digestible energy concentration of the diet and promote the digestion and absorption of nutrients and minerals by livestock. High-fiber forage increases the rate of passage in the gastrointestinal tract, resulting in a reduction in nutrient absorption and utilization rates, and deterioration of feed efficiency. A fiber content that is too low will also lead to difficulty in reaching satiety for livestock, which may even cause constipation (Gu et al., 2021). Therefore, it is necessary to regulate the crude fiber content reasonably. The crude fiber content measured in this experiment (7.62%) was lower than that of mulberry leaves (11.1%) (Wu et al., 2017), *Paulownia paulownia* (9.83%) (Wu et al., 2017), and other woody diets. In the present study, the crude fiber content was moderate, similar to that of *C. aconitifolius* (8.54%) (Gu et al., 2021) and *E. ulmoides* (6.87%) (Wang et al., 2017). Therefore, the moderate fiber content of *P. tatarinowii* leaves can prevent side effects that occur in forage with a fiber content that is too high or too low, thus improving digestibility. Crude fat in plants is generally regarded as a representative of energy, and a higher crude fat content indicates higher feed energy. The crude fat content measured in this study (6.79%) was slightly higher than the dietary requirement of 20–50 g·kg^−1^ for ruminants (Li et al., 2006), which may have affected the digestion of nutrients in the rumen. Other low-fat diets can be used to adjust the feed. Calcium (Ca) and phosphorus (P) are two important elements in the mineral nutrition of domestic animals, and play a special role in development and maintenance of domestic animals. In this experiment, the calcium (58.01 g·kg^−1^) in the leaves was significantly higher than that of phosphorus (0.785 g·kg^−1^), which may have been caused by the main growth environmental factors. *P. tatarinowii*, which inhabits limestone areas, is commonly used as a calcite soil indicator plant, and has a higher Ca absorption capacity than that of P. Therefore, the soil quality and fertilization ratio can be properly adjusted to improve the calcium to phosphorus ratio when this species is planted as a woody forage species.

Studies have shown that small amounts of tannins can have an astringent role in the digestive tract of animals, with a slight antidiarrheal effect, increasing the degradation rate of protein in the rumen, and improving the digestibility of dry matter (Puchala et al., 2005). Robbins et al. (1987) and Jackson et al. (1996) studied tannin contents in the leaves of tropical trees, shrubs and legumes and found that plants with tannin contents less than 55 g·kg^−1^ (dry mass) could be used as animal feed. However, when the tannin content reaches 60–90 g·kg^−1^ (dry mass), animals generally refuse to consume the leaves. The average tannin content measured in this experiment was 4.97 g·kg^−1^, and the highest was only 5.59 g·kg^−1^, which is far lower than the antifeedant standard. Simultaneously, the low tannin content improved the dry matter digestibility of *P. tatarinowii* leaves to some extent.

The average total amounts of hydrolyzed and free amino acids were 108.20 mg·g^−1^ and 47.87 mg·g^−1^, respectively, and both contained eight essential amino acids. The sums of essential amino acids (E) in the hydrolyzed amino acids in the three samples were 124.71 mg·g^−1^, 110.17 mg·g^−1^, and 89.73 mg·g^−1^. The sums of the non-essential amino acid contents (N) were 32.21 mg·g^−1^, 30.38 mg·g^−1^, and 27.57 mg·g^−1^, respectively. The E/T values were 0.74, 0.72, and 0.69, respectively, and the E/N values were 2.87, 2.63, and 2.25, respectively. All those values are close to or consistent with the reference protein model proposed by WHO/FAO, which states that E/T should be 0.40 and E/N should be above 0.6 (FAO, 1970). Moreover, the forage indices of NDP, ADP, DMI, DDM, and RFV in 21 populations all met the super standards of the American Grass and Grassland Association (AFGC) for hay.

In addition, based on the characteristics of *P. tatarinowii*, including its poor drought tolerance, developed root system, strong tillering ability, and long life, the advantages of planting *P. tatarinowii* include extensive management and many years of benefits from one planting. At the same time, *P. tatarinowii* has strong adaptability, and is mainly distributed in limestone areas with poor site conditions and rich resources in 19 provinces in China. Thus, the planting and feeding types of *P. tatarinowii* can be used to afforest barren hills, improve water and soil retention, and prevent them from occupying arable land.

In summary, *P. tatarinowii* has the material basis for the development into an excellent woody forage tree, with high crude protein, crude fat, and calcium content, low fiber and tannin content, and a protein-rich composition. Therefore, this species has specific prospects for development and utilization.

### Geographical variation trend of leaf forage characteristics of *P. tatarinowii* populations

Variability is the result of different environmental selection forces, which are the root cause of physiological differentiation among populations. In this study, the results of the correlation analysis showed that there was no significant correlation between forage characteristics and latitude and longitude, indicating no significant geographical variation. There was a strong correlation between forage characteristics and altitude, average annual temperature, and precipitation, indicating that high altitude, low temperature, and rainy weather could improve the forage value of leaves. The same trend was reported by Dewhurst and King (1998), and Darwish (2014), who found higher oil yields and higher protein content in plants collected from relatively higher elevations. However, genetic factors cannot be ignored, and plant morphology and physiological indicators are affected by both genetic and environmental factors (Rahimmalek et al., 2017). It is necessary to consider differences within and among populations when screening for promising germplasm resources. Thus, further research should be conducted to explore the effects of the environment and genotypes when selecting genotypes with a high protein content.

The results of the cluster analysis showed that the forage characteristics of *P. tatarinowii* had significant regional characteristics, and these characteristics could be divided into four groups: the Yangtze River Basin, northern, southwestern, and eastern coastal populations. The populations in the north and southwest did not overlap, but the populations in the Yangtze River Basin and the eastern coastal areas overlapped extensively and frequently transmitted genetic information. It was speculated that the seeds of *P. tatarinowii* moved from the river and gradually took root and developed into multiple populations; therefore the populations in this region were closely related. Our future research direction will be to detect and analyze the genetic diversity of *P. tatarinowii*, verify its genetic relationship at the molecular level, and explore its origin and evolution. The results also suggest that when further investigating and protecting *P. tatarinowii* germplasm resources, we could focus on classification, protection, and collection according to region. At the same time, we should allocate and plant this species according to the specific region to improve adaptability, increase survival rates, and reduce costs. *P. tatarinowii* has the advantage of strong tillering, and its biomass can be improved by continuously cutting and promoting lateral branches. Therefore, screening excellent populations and breeding excellent varieties with high biomass and nutritional value are among the next research directions.

### Comprehensive evaluation and screening of *P. tatarinowii* population with superior forage

A high CP content and low NDF and ADF content in forage are important indicators of high nutritional value (Papachristou and Papanastasis, 1994; Li et al., 2006). Based on this information, SDJN and AHXX were two high-protein content populations that met the grade 1 standard; thus, they were screened out. Two high-quality forage populations, SDZZ and SXLQ, were selected according to the forage evaluation indices NDF, ADF, DMI, DDM, and RFV. Among the four high-quality populations, three are northern populations, and one borders the northern region (AHXX is in the northern part of the Anhui Province and belongs to the northern region). In conclusion, from the point of view of the leaf forage characteristics of *P. tatarinowii*, in comparison with the other areas, the northern cool areas represented by Shandong, Shanxi and northern Anhui are more suitable for planting, which is consistent with the results of the correlation analysis. The correlation analysis results showed that cold and cool areas at a specific altitude should be selected as the distribution area for planting *P. tatarinowii* for leaf forage purposes.

## Conclusion

In summary, the leaves of *P. tatarinowii* in this study had high crude protein, crude fat, and calcium contents, low fiber and tannin contents, and were protein-rich; thus, this species has the material basis to be developed into an excellent woody forage tree species. Four high-protein and high-RFV feeding populations, SDJN, SDZZ, SXLQ, and AHXX, were selected according to forage evaluation indices. The results of the correlation analysis showed that there was no significant correlation between forage characteristics and latitude and longitude, indicating no significant geographical variation. However, forage characteristics were strongly correlated with altitude, average annual temperature, and annual precipitation, indicating that high altitude, low temperature, and rainy conditions could improve the forage value of leaves. *P. tatarinowii* can be planted to provide leaf forage in cool and wet areas at a specific elevation. Moreover, the forage value of *P. tatarinowii* leaves can be further improved by increasing nitrogen fertilizer and reducing K and Ca fertilizers during cultivation. The forage characteristics of *P. tatarinowii* showed obvious regionalism, and the populations were divided into four groups: the Yangtze River Basin and northern, southwestern, and eastern coastal populations. In future studies, to support the protection of *P. tatarinowii* germplasm resources, populations should be classified and collected according to region.

## Data availability statement

The original contributions presented in the study are included in the article/**Supplementary Material**. Further inquiries can be directed to the corresponding author.

## Author contributions

YG and QQ participated in the experiments and the writing of the paper. T-TC, C-XZ, and YY analyzed the data. LZ, Q-ZL, and YL provided revisions and contributed constructive suggestions. QQ and YG designed the experiment and oversaw writing of the paper. All authors contributed to the article and approved the submitted version.
